# Two Component Systems: Physiological Effect of a Third Component

**DOI:** 10.1371/journal.pone.0031095

**Published:** 2012-02-17

**Authors:** Baldiri Salvado, Ester Vilaprinyo, Hiren Karathia, Albert Sorribas, Rui Alves

**Affiliations:** 1 Departament de Ciències Mèdiques Bàsiques, Universitat de Lleida & IRBLleida, Lleida, Spain; 2 Evaluation and Clinical Epidemiology Department, Parc de Salut Mar and CIBER of Epidemiology and Public Health (CIBERESP), Barcelona, Spain; Center for Genomic Regulation, Spain

## Abstract

Signal transduction systems mediate the response and adaptation of organisms to environmental changes. In prokaryotes, this signal transduction is often done through Two Component Systems (TCS). These TCS are phosphotransfer protein cascades, and in their prototypical form they are composed by a kinase that senses the environmental signals (SK) and by a response regulator (RR) that regulates the cellular response. This basic motif can be modified by the addition of a third protein that interacts either with the SK or the RR in a way that could change the dynamic response of the TCS module. In this work we aim at understanding the effect of such an additional protein (which we call “third component”) on the functional properties of a prototypical TCS. To do so we build mathematical models of TCS with alternative designs for their interaction with that third component. These mathematical models are analyzed in order to identify the differences in dynamic behavior inherent to each design, with respect to functionally relevant properties such as sensitivity to changes in either the parameter values or the molecular concentrations, temporal responsiveness, possibility of multiple steady states, or stochastic fluctuations in the system. The differences are then correlated to the physiological requirements that impinge on the functioning of the TCS. This analysis sheds light on both, the dynamic behavior of synthetically designed TCS, and the conditions under which natural selection might favor each of the designs. We find that a third component that modulates SK activity increases the parameter space where a bistable response of the TCS module to signals is possible, if SK is monofunctional, but decreases it when the SK is bifunctional. The presence of a third component that modulates RR activity decreases the parameter space where a bistable response of the TCS module to signals is possible.

## Introduction

Two component systems (TCS) are biochemical signaling modules that are ubiquitous in bacteria and are also present in some eukaryotes. Prototypical TCS are composed of two proteins: a sensor kinase (SK) and a response regulator (RR). The SK phosphorylates a histidine residue and subsequently transfers the phosphate to an aspartate residue in the RR. There are many variations around this prototype, ranging from phosphorelays that can concatenate up to three phosphotransfers (His→Asp→His→Asp) between different proteins to hybrid kinases in which the SK and the RR domains are fused in the same protein [1], [2]. In prototypical TCS, the SK can be ***bifunctional*** if, when unphosphorylated, it increases the dephosphorylation rate of the RR. Otherwise, the SK is ***monofunctional***. The majority of well characterized SKs are bifunctional, with a few, such as the chemotaxis regulating CheA, being monofunctional.

In addition to SKs and RRs, some TCS are also known to interact with specific phosphatases that regulate dephosphorylation of the RR [3]. These core components of TCS and phosphorelays are also complemented by auxiliary proteins that play a regulatory role in the activity of some TCS, transmitting the cognate signal to the SK. For example, the SK CheA is regulated through its interaction with membrane receptors that detect chemical compounds in the medium and direct organisms towards higher concentrations of nutrients [4] and the activity of the SK NRII that regulates nitrogen fixation is modulated through its interaction with the protein PII [5].

In recent years, interactions between the TCS and auxiliary proteins were identified as a strategy to integrate non-cognate signals in the regulation of TCS [6]. For example, the orphan SK RetS interacts with the GacS SK, preventing the response of the latter to its cognate signal [7], [8], [9], [10], [11] and the peptide PmrD binds to and protects the phosphorylated form of the RR PmrA from the phosphatase activity of its cognate SK, PmrB [12]. The GacS/GacA TCS regulates virulence in *Pseudomonas aeruginosa* [13], [14], while the PmrB/PmrA TCS is required for resistance of *Salmonella* to acidic and antibiotic stresses, among others [12], [15]. These systems raise the question of understanding the effect of such interactions with the core TCS module in the operating regime of the module and what consequences these effects may have on the influence of the module on the cellular physiology of the organism [16], [17], [18], [19], [20], [21], [22]. Previous studies suggested that a third component that binds to and protects the phosphorylated form of the RR causes delays in the response of autogenous TCS that regulate their own expression [12], [17], [22]. However, to our knowledge, no studies were made about the effect that binding of a third component to the SK has on the potential dynamic behavior of the TCS module. In addition, the effect of both types of third component proteins were not studied in TCS that do not regulate their own expression.

In previous work we have used mathematical models to characterize the effect of diverse architectures on the signaling response of prototypical TCS. The analysis of such models enables understanding if particular physiological responses are more effectively achieved by one of several alternative designs of the network that executes the biological process of interest [23]. Such studies are difficult, if not impossible to do without the assistance of those mathematical models. In the case of the TCS, we showed that TCS with bifunctional SKs are more effective in buffering the TCS against crosstalk, while monofunctional SK are more effective in integrating different signals [24], [25]. We have also identified necessary conditions for the existence of post-translational bistable responses in prototypical TCS [25]. If a system is capable of bistable responses, this means that its output variable can assume one of two possible values as a consequence of the same input. The specific value that the variable assumes depends on the value that the variable had before the stimulus. Post-translational bistability is only possible in TCS in which the affinity between the phosphorylated SK and unphosphorylated RR is similar to that between the unphosphorylated forms of the proteins. In addition, a large fraction of the dephosphorylation flux of the RR must be independent of any phosphatase activity of the SK [25].

Given these considerations, in this work our goal is to understand the physiological effect of a third protein, such as RetS or PmrD, on the function of canonical TCS in the absence of auto-regulation of gene expression. To achieve this, we built and analyzed mathematical models for the alternative designs of TCS with and without such a third component, and compared the dynamic behavior of the different systems. This analysis identifies specific physiological behaviors that are more effectively executed by each alternative design for the TCS.

Our study reveals that a RR-binding third component (TC_RR_) decreases the region in parameter space where a bistable response is possible, while a SK-binding third component (TC_SK_) increases the parametric region where a bistable response is possible when the SK is monofunctional and decreases it when the SK is bifunctional.

## Results

**Figure 1:**
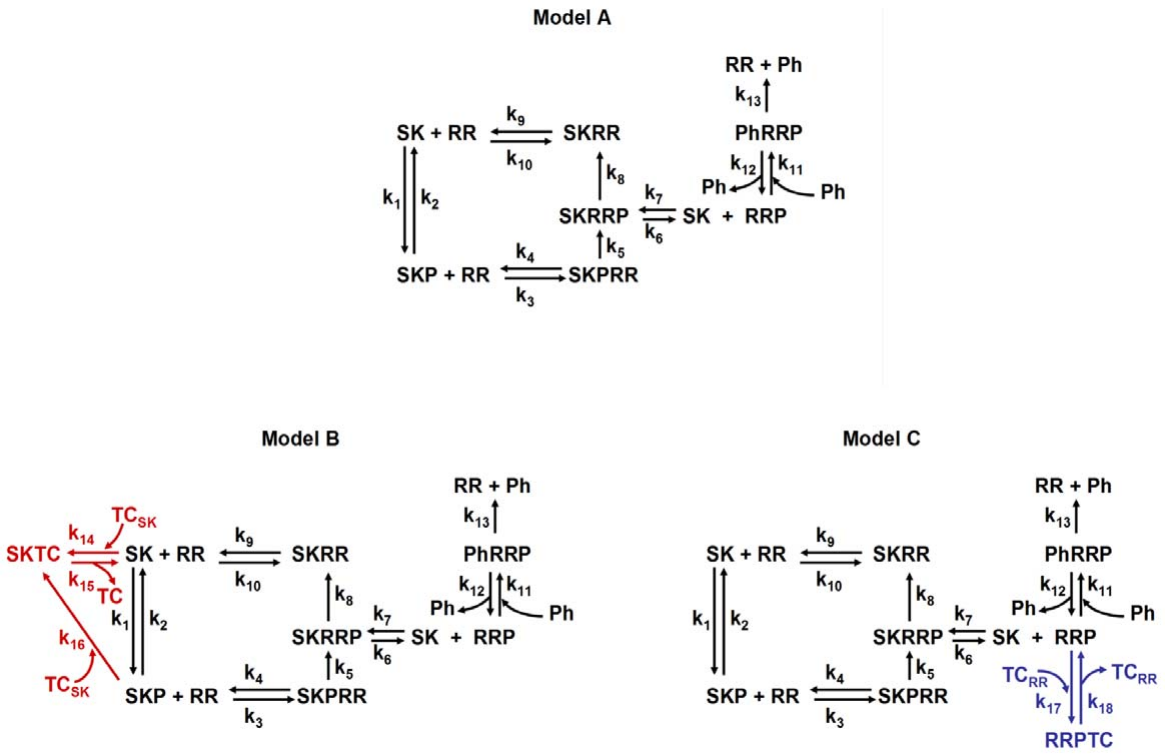
Analyzed Two Component Systems modules. Model A represents a prototypical TCS. Model B represents a TCS with a SK-binding third component (TC_SK_). Model C represents a TCS with a RR-binding third component (TC_RR_). SK: sensor kinase; RR: response regulator; SKP: phosphorylated SK; RRP: phosphorylated RR; Ph: alternative phosphatase that dephosphorylates RRP; SKRR: dead-end complex, resulting from the binding of SK and RR; SKPRR: protein complex formed by the binding of SKP and RR; SKRRP: protein complex formed by the binding of SK and RRP; PhRRP: protein complex formed by the binding of Ph and RRP; SKTC and RRPTC: protein complexes formed by the binding of the third component to SK and RRP, respectively; (k_1_, …, k_18_): kinetic constants of the individual reactions. For simplicity, ATP and the release of inorganic phosphate are omitted. To analyze TCS modules with monofunctional sensors, k_8_ is set to 0. To analyze TCS modules with bifunctional sensors, k_8_ is set to be different from 0.

In order to understand the physiological effect of a third component (TC) on the function of a prototypical TCS, we built models of TCS with and without that TC and compared the dynamical behavior of those models. Figure 1 shows a schematic representation of the three models used in our analysis. These models are mathematically described by using a mass action system of ordinary differential equations (ODE) [26]. The resulting ODE systems for each of the three alternative models can be analyzed and compared numerically by running appropriate simulations on a computer.

### Models and Comparisons

The network model that we use to describe the prototypical TCS in our analysis is that defined in Igoshin *et al.* [25], which is based on earlier work [27]. In Model A, shown in Figure 1, the SK can autophosphorylate and/or autodephosphorylate in response to an external signal. Both phosphorylated and unphosphorylated forms of SK are allowed to bind RR with similar affinities, as reported in [28], [29], [30]. Binding of unphosphorylated SK and RR is reversible and forms a dead-end complex (SKRR). Phosphorylated SK (SKP) can transfer its phosphate to the RR. The phosphorylated RR (RRP) will modulate the biological levels and activity of relevant proteins.

This network for the prototypical TCS was modified to study the effect of a TC binding to either the SK or the RR. The changes in the network are also shown in Figure 1. Model B represents a TCS where a third component binds to the SK (TC_SK_), inactivating it. Model C represents a TCS where a third component binds to the phosphorylated RR (TC_RR_) and stabilizes this phosphorylated form. In prototypical TCS modules with ***bifunctional sensors***, the unphosphorylated SK can destabilize the phosphorylated form of the RR and it increases the dephosphorylation rate of RRP (k_8_>0 in Figure 1). In prototypical TCS modules with ***monofunctional sensors***, the unphosphorylated SK has no effect upon the dephosphorylation rate of RRP (k_8_ = 0 in Figure 1). The model includes a phosphatase that dephosphorylates RRP independently of the SK. This is done for generality. In the cases where no such phosphatase exists, this set of reactions can be replaced by a single reaction where the unstable RRP phosphate bond hydrolyzes over time. An appropriate choice of parameter values will make the results of the analysis similar to those described for the full model.

In this study we analyze the potential effect of a TC in the physiological behavior of TCS modules with bifunctional and monofunctional sensors independently. If the TC has no effect on the physiological behavior of the TCS, then the presence of TC in particular instances of TCS should be understood as an evolutionary accident. If the TC has an effect on the physiological behavior of the TCS, this could provide a rationale for the selection of a TCS design that includes a TC. To perform the analysis, we compare the dynamical behavior of Model A to that of Models B or C, independently. This comparison is done in two ways.

First, Models A and B (or C) are compared ensuring that the parameter values of all processes that are common are the same in the two models. This guarantees that whatever differences are found are only due to the addition of the TC. This comparison is equivalent to comparing an organism where the TCS interacts with a TC to another where the TC has been deleted from the genome. This situation could occur, for example during the creation of a new biological circuit by genetic manipulation in a biotechnological context. ***Thus, this type of comparison is relevant for understanding the differences in behavior of biological circuits created using synthetic biology techniques***.

Second, we also perform a mathematically controlled comparison between Models A and B (or C). This is a well established method for evaluating the irreducible effect of a change in the design of a biological circuit on the dynamic behavior of the network [31]. In this comparison, in addition to ensuring that Models A and B (or C) have the same values for corresponding parameters of all processes that are common, we use the differences between the designs as degrees of freedom that evolution can use as a substrate to minimize differences between the dynamic behavior of the two systems. If the alternative designs can be made equivalent by using these degrees of freedom, then one may argue that they cannot be distinguished by natural selection. If, after making the systems as equivalent as possible, there are still irreducible differences in the physiological behavior between designs, then one may expect one of them to be preferably selected when its functionality provides better adaptive advantage. In the models under comparison, the difference is the deletion of a protein from the module between Model B (or C) and Model A. In this situation, the protein burden caused by Model A is lower than that caused by its alternative designs. Hence, we allow that the system changes the total concentrations of the remaining proteins (SK and/or RR). The details for this comparison are given in the methods section. ***This comparison is thus relevant for understanding the differences in the dynamic behavior that are intrinsic to the differences in design between Models A and B (or C), and to those alone, in evolutionary terms.***

### Effect of a third component on TCS signal amplification and bistability

**Figure 2:**
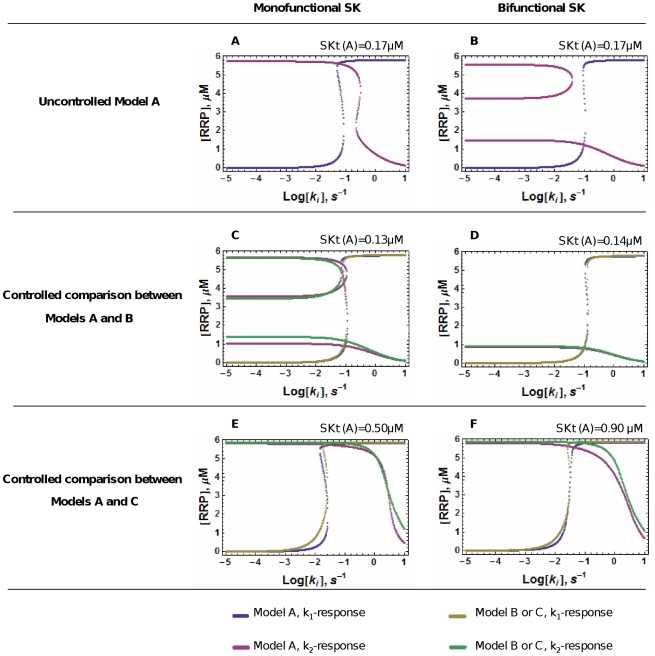
Steady state signal-response curves for the various TCS modules. Each plot shows the steady state levels of the phosphorylated RR in the y axis at different values of the signal k_1_ (SK autophosphorylation rate constant) or k_2_ (SKP dephosphorylation rate constant) in the x axis. When the signal modulates SK dephosphorylation (changes in k_2_), the system behaves symmetrically to when SK phosphorylation (changes in k_1_) is modulated. In the first case, increases in signal intensity cause the fraction of RRP to decrease, while in the latter, increases in signal intensity cause the fraction of RRP to increase. A, C, E: Response curves of TCS modules with monofunctional sensor. B, D, F: Response curves of TCS modules with bifunctional sensor. A, B, Response curves of Model A. C, D: Mathematically controlled comparison between the response curves of Model B and those of Model A. E, F: Mathematically controlled comparison between the response curves of Model C and those of Model A. Mathematical controls are implemented to make sure that the differences in response between the alternative modules are due to the presence of third component and not to other spurious differences.

Signal amplification is an important physiological property of TCS. TCS with appropriate signal amplification can provide evolutionary advantages to organisms harboring them. Thus, understanding how signal amplification is affected by adding a TC to a TCS would help in predicting under which conditions to expect such a design to be selected. Figure 2 shows that all models can achieve the same signal amplification, whether the environmental signal modulates the autophosphorylation (k_1_) or the autodephosphorylation (k_2_) of the SK. This can be seen because the difference between the amount of RRP (phosphorylated RR) when k_1_ is low (k_2_ is high) and when k_1_ is high (k_2_ is low) can be similar for all models. Nevertheless, Model B responds at higher signal intensities and Model C responds at lower signal intensities than Model A, when the stimulus modulates the SK autophosphorylation reaction rate (compare the curves for k_1_ response of Model A to those of Models B and C in Figure 2). When the signal modulates the SK autodephosphorylation reaction rate, Model B responds at lower signal intensity and Model C at higher signal intensity than Model A (compare the curves for k_2_ response of Model A to those of Models B and C in Figure 2). However, mostly, the differences in signal intensity at which the systems are turned ON or OFF are small.

In addition, the prototypical TCS shown in Model A can show bistable behavior [25], making it possible that a signal can lead to one of two alternative responses, depending on the history of the system. Such a response may have some evolutionary advantages, for example in situations like sporulation where an irreversible developmental decision is made by cells. Bistable regions in the curves of Figure 2 have three values of RRP for a single value of signal intensity. The two extreme values are the alternative stable steady states, while the middle value is a biologically irrelevant unstable steady state that is mathematically required to exist if two stable steady states are present. In the figure one can see that the signaling ranges where bistability exists are different if the environmental signal modulates the autophosphorylation (k_1_) or the autodephosphorylation (k_2_) of the SK.

Necessary, although not sufficient, conditions for the existence of such bistable behavior in the prototypical TCS are i) the formation of a dead-end complex between the dephosphorylated forms of SK and RR and ii) that a sufficiently high fraction of the flux for the dephosphorylation of RRP is independent of SK. To understand how the presence of a TC affects the possibility of a bistable response in the prototypical TCS, we analyzed Models B and C in search of the existence of multiple steady states, followed by a comparison of the physiological behavior between Models A and B, and between Models A and C.

Given that signals can in principle modulate either the autophosphorylation (k_1_) or the autodephosphorylation (k_2_) rate of SK, we performed parallel computational experiments independently modulating their intensity. These experiments were done independently for models with monofunctional and bifunctional SK (Figure 2).

Our results show that, in an uncontrolled comparison, the range of bistability for the bifunctional prototypical TCS is larger than if a TC binds any of the proteins of the module (compare panel B to panels D and F of Figure 2). Bistability for Model B in panel D is only observed for k_1_ signaling, while no bistability is observed for Model C in panel F. On the other hand, the range of bistability for the monofunctional prototypical TCS is larger than if a TC binds the RR of the module (compare panel A to panel E of Figure 2), but smaller than if the TC binds the SK (compare panel A to panel C of Figure 2). Differences among the three systems are more pronounced when the signal induces dephosphorylation of the SK (k_2_), rather than inducing SK autophosphorylation (k_1_).

An additional definition is needed before further presenting and discussing the results. Hereafter the system is said to be in an ON state if most of its RR is in the phosphorylated RRP form. If most of the RR is in its dephosphorylated form, the system is said to be in its OFF state. With this in mind, and as one might expect, systems with a TC_SK_ are in an ON state for a smaller signaling range (panels C and D) and systems with a TC_RR_ are in an ON state for a larger signaling range (panels E and F), in comparison with the uncontrolled Model A (panels A and B).

When the comparisons are controlled we see that the response of Model A can become similar to that of Model B or C by adjusting the total amount of available SK. If the response of Model B is to be mimicked, the total amount of SK in Model A is decreased (Figure 2, panels C and D, see methods for the exact values of the total amount of SK), while mimicking the response of Model C leads to an increase in the concentration of SK (Figure 2, panels E and F, see methods for the exact values of the total amount of SK).

The k_2_-response curves in Figure 2 panels B and C show that the switch from ON to OFF (from high to low levels of RRP) in these models could be irreversible or very difficult to reverse. In other words, modulation of the autodephosphorylation rate of SK by an external signal could generate nearly irreversible biological switches.

Our simulations also show that the necessary conditions for bistability in prototypical TCS remain necessary in the TCS with a TC. If either no independent phosphatase is present in the system (Ph = 0) or no dead end complex is formed (k_10_ = 0) all TCS modules analyzed here are monostable (see section “Effect of changes in SK-independent RRP dephosphorylation and SKRR affinity on bistability” below).

In summary, a TC_RR_ causes a reduction in the TCS parameter space of bistability and an increase in the signaling range in which the system is in the ON state (responds at lower k_1_-signal intensity and at higher k_2_-signal intensity), whether the SK is monofunctional or bifunctional. This can be more effectively compensated by prototypical TCS through a change (an increase) in the concentration of the SK. In contrast, TC_SK_ increases the signaling range in which the TCS can show a bistable response if and only if the SK is monofunctional and the environment modulates k_2_ (SK dephosphorylation rate). The behavior of TCS with a TC_SK_ can be mimicked by prototypical TCS through a change (a decrease) in the concentration of the SK.

### Effect of a third component on TCS response time

In addition to signal amplification, the response time to signals is an important physiological property of TCS. In evolutionary terms, a change in response time may have important consequences to the fitness of the system. Therefore, we analyzed the effect of a TC on the response times of the TCS. To do this we performed four independent sets of experiments for each of the models, and independently considering systems with a monofunctional SK and with a bifunctional SK. In experiments 1 and 2 we instantaneously change the signal k_1_ and measure how long the system takes to come within 90% of its new steady state. This measures the response time of the system if the physiological signal modulates SK phosphorylation. In experiments 3 and 4, we instantaneously change the signal k_2_ and measure how long the system takes to come within 90% of its new steady state. This measures the response time of the system if the physiological signal modulates SK dephosphorylation. The details about how the experiments were run are as follows:

1 - We set each system to its OFF state, with k_1_ = 10^−5^ s^−1^. Then, we increased the value of k_1_ to a value k_1 higher_ and measured how long the system took to get to within 90% of its new steady state value. k_1 higher_ was systematically changed between 10^−5^ and 10 s^−1^.
2 - We set each system to its ON state, with k_1_ = 10 s^−1^. Then, we decreased the value of k_1_ to a value k_1 lower_ and measured how long the system took to get to within 90% of its new steady state value. k_1 lower_ was systematically changed between 10^−5^ and 10 s^−1^.
3 - We set each system to its OFF state, with k_2_ = 10 s^−1^. Then, we decreased the value of k_2_ to a value k_2 lower_ and measured how long the system took to get to within 90% of its new steady state value. k_2 lower_ was systematically changed between 10^−5^ and 10 s^−1^.
4 - We set each system to its ON state, with k_2_ = 10^−5^ s^−1^. Then, we increased the value of k_2_ to a value k_2 higher_ and measured how long the system took to get to within 90% of its new steady state value. k_2 higher_ was systematically changed between 10^−5^ and 10 s^−1^.

**Figure 3:**
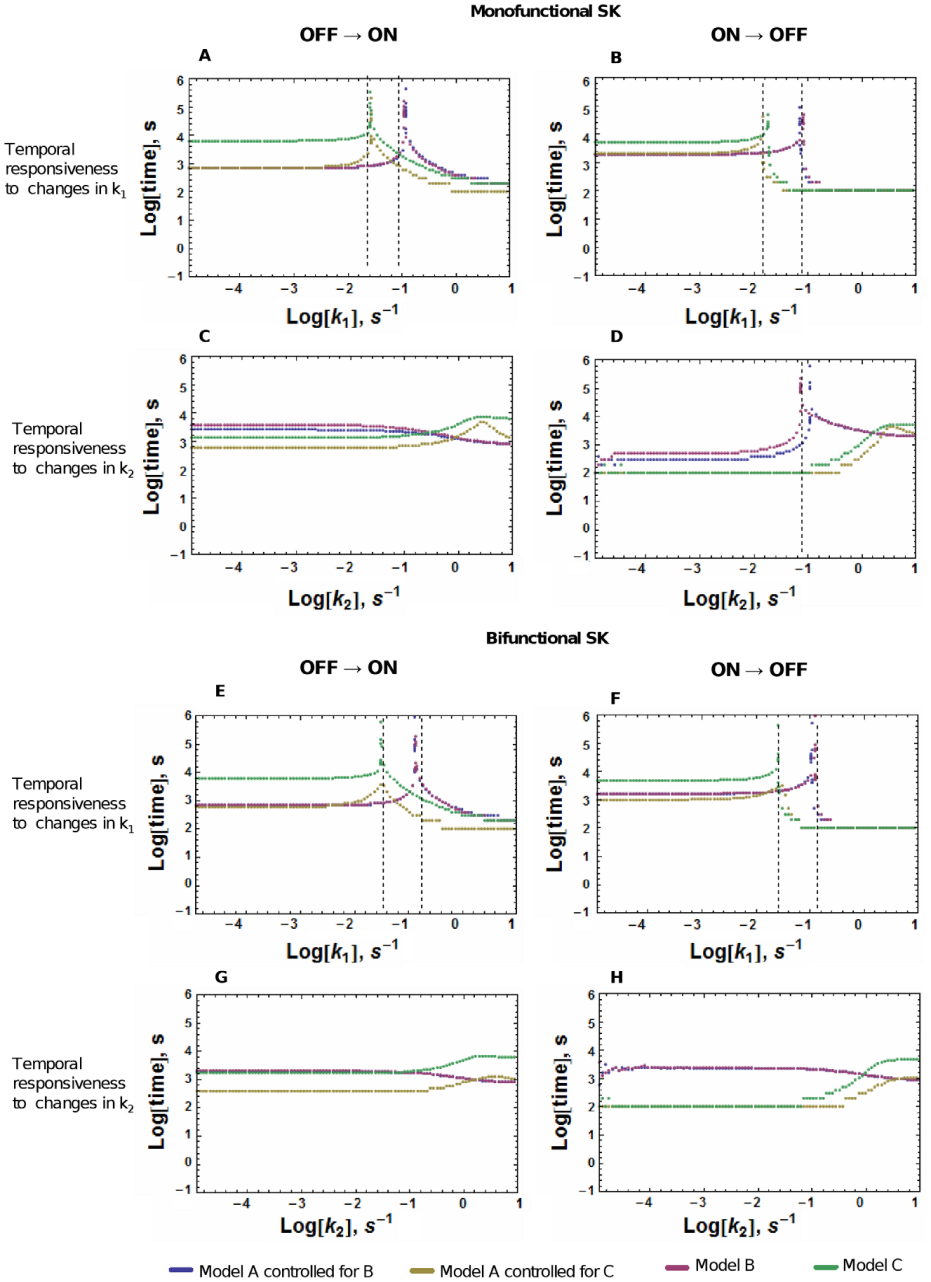
Temporal responsiveness curves of Models A, B, and C. The systems are at an initial steady state and, at time zero, the signal, represented in the x axis, changes instantaneously and the time it takes for the system to get to within 90% of the new steady state is measured and plotted in the y axis. A–D: Response times of TCS with monofunctional SK. E–H: Response times of TCS with bifunctional SK. The OFF to ON plots start with the systems at an OFF steady state (low levels of RRP) corresponding to a low value of k_1_ (A, C, E, G) or a high value of k_2_ (B, D, F, H). The signal is then changed to increase the steady state level of RRP. The ON to OFF plots start with the systems at an ON steady state (high levels of RRP) corresponding to a high value of k_1_ or a low value of k_2_. The signal is then changed to decrease the steady state level of RRP. Peaks that indicate slower response times are located immediately outside the range of bistability. The lack of a peak in a curve can be due to monostability or irreversibility. The dashed lines indicate the signal value at which Models B and C exit its bistable range. Absence of a dashed line indicates irreversible turning ON or OFF of the system (Model B in panel C ) or absence of bistability (see the signal-response curves of Figure 2).

Results are shown in Figure 3. We see that the response times increase by more than two orders of magnitude when the new parameter value k_·lower_ or k_·higher_ approaches the threshold value for exiting the bistability region of a system. The peaks of slower response in the curves in Figure 3 are in the region of signal intensity that lies immediately beyond the border of the bistability ranges shown in Figure 2. Given that the peaks of slower response are located at the exit of the bistable region, there is no peak in the signal-response time curve when the response is monostable or when there is an irreversible turning OFF of the system. Model B and Model A|B (A controlled for B) don't have a peak in their OFF to ON k_2_-response times (Panel C of Figure 3) because these models irreversibly turn OFF after an increase in k_2_ (as depicted in Figure 2 panel C). Model C also has no peak in the response time (Panels C and D of Figure 3) because this model has a monostable response to changes in k_2_ (see Figure 2 panel E). In panels G and H of Figure 3, neither of the three systems shows a peak in their signal-response time curve because of the lack of bistability in their signal-response steady state curve (see Figure 2 panels D and F). When Model A is compared to Model B in an uncontrolled manner, the time response peaks of Model A appear at signal intensities that are always lower than those where the peak appears in the response of Model B. When Model A is compared to Model C in an uncontrolled manner, the time response peaks of Model A appear at signal intensities that are always higher than those where the peak appears in the response of Model C (see Figure S1).

In order to have a proxy of the integral temporal responsiveness of each system, we calculated the area under each of the signal- response time curves shown in Figure 3. This area is the sum of all the transient response times for each signaling response. The values of these areas are given in Table 1 and show that overall response times are similar between Models A and B. In contrast, Model A has a faster response than Model C. When the comparison is not controlled, differences between integrated response times of the three models are small, when the signal modulates autophosphorylation of SK. However, if SK dephosphorylation is modulated, Model B has the fastest integrated response, followed by Model A. Model C is, again, the slowest responder (Table S1).

**Table 1:** Controlled comparison of the overall response times between Models A and B, and between Models A and C^a^.

	Modulation of SK autophosphorylation (k_1_)		Modulation of SKP dephosphorylation (k_2_)	
	OFF→ON	ON→OFF	OFF→ON	ON→OFF
**Monofunctional**				
Model A|B	3 646.18	1 244.27	9 129.47	24 524.50
Model B	3 406.48	1 337.95	9 467.02	24 801.00
**Bifunctional**				
Model A|B	3 917.63	1 501.14	8 656.10	10 565.20
Model B	3 672.27	1 739.08	8 695.38	10 672.20
**Monofunctional**				
Model A|C	1 351.02	1 003.90	21 984.30	26 656.70
Model C	3 125.05	1 091.73	57 574.80	43 048.20
**Bifunctional**				
Model A|C	1 152.38	1 029.89	10 647.20	8 972.97
Model C	3 358.06	1 195.35	57 212.80	40 114.40

a The reported values represent the area below each curve in Figure 3, that is, the sum of the transient times for each response. A|B stands for Model A controlled for Model B. A|C stands for Model A controlled for Model C.

In summary, Model B is a faster overall responder than the prototypical TCS when the system is turned ON by modulating the phosphorylation rate of the SK, and it is a slower responder in any other case. In contrast, Model C is always slower to turn ON or turn OFF than the prototypical TCS, under controlled comparison conditions.

### Stochastic effects of a third component

Fluctuations in the amount of proteins that participate in biological reactions can lead to stochastic effects in the system's behavior, when the total number of proteins participating in reactions is small. We performed stochastic simulations to understand the role of stochasticity on the effect of the TC on the physiological response of the TCS networks. These simulations take into account that the number of TCS proteins present in the cell are typically in the 10–1000 molecules range.

**Figure 4:**
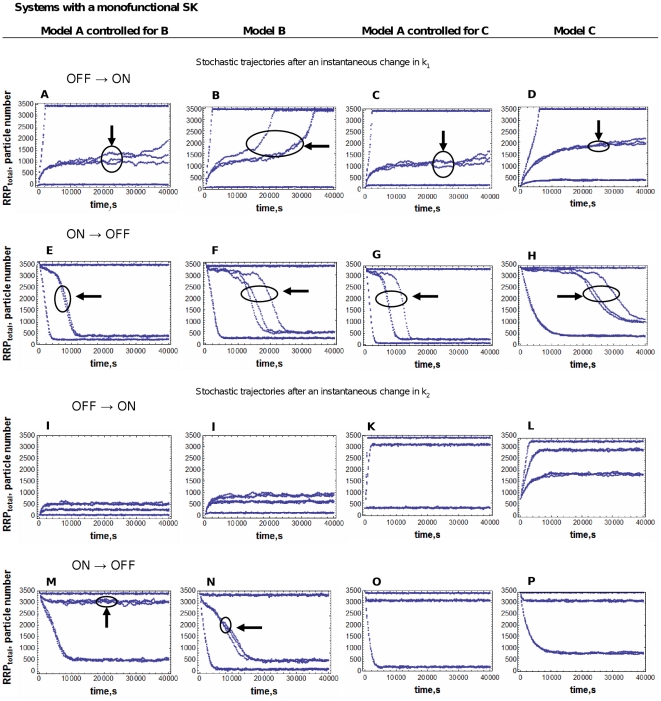
Stochastic time trajectories after an instantaneous change in the signal, for the three systems modeled with a monofunctional SK. A mathematically controlled comparison between Models A and B, and between Models A and C was performed as described in methods. The results for three individual runs for each value of k1 or k2 are plotted in each panel. Panels in the first column correspond to Model A controlled to be as similar as possible to Model B. Panels in the second column correspond to Model B. Panels in the third column correspond to Model A controlled to be as similar as possible to Model C. Panels in the fourth column correspond to Model C. The circles indicate lines that are replicates of the same simulation. Simulations marked with an arrow correspond to a signal intensity close to the bistability threshold and show slower and noisier responses. The OFF to ON plots start with the systems at an OFF steady state (low levels of RRP) corresponding to a low value of k1 or a high value of k2. At time zero, there is an instantaneous increase in k1 or decrease in k2. The ON to OFF plots start with the systems at an ON steady state (high levels of RRP) corresponding to a high value of k1 or a low value of k2. At time zero, there is an instantaneous decrease in k1 or increase in k2. The values for k1 or k2 are chosen to be below, next to and above the threshold value at which the system switches from OFF to ON, or from ON to OFF. See text for further details.

**Figure 5:**
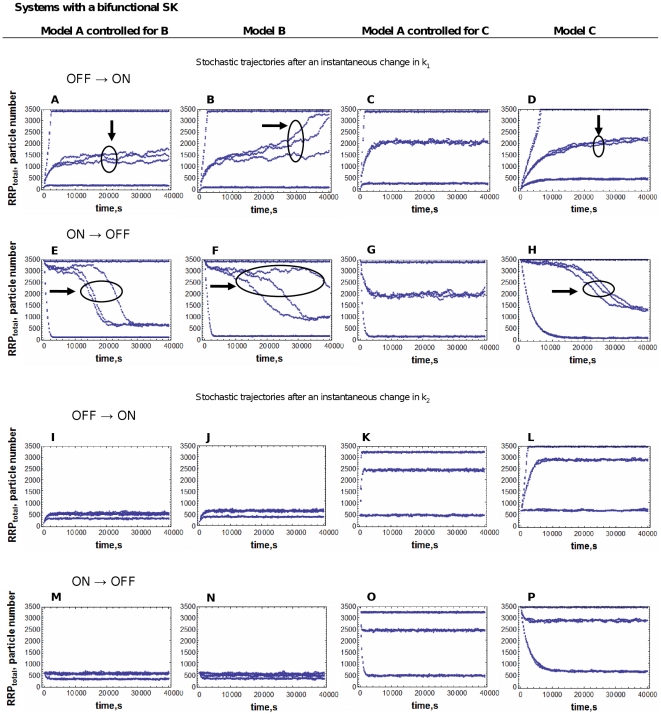
Stochastic time trajectories after an instantaneous change in the signal, for the three systems modeled with a bifunctional SK. A mathematically controlled comparison between Models A and B, and between Models A and C was performed as described in methods. The results for three individual runs for each value of k1 or k2 are plotted in each panel. Panels in the first column correspond to Model A controlled to be as similar as possible to Model B. Panels in the second column correspond to Model B. Panels in the third column correspond to Model A controlled to be as similar as possible to Model C. Panels in the fourth column correspond to Model C. The circles indicate lines that are replicates of the same simulation. Simulations marked with an arrow correspond to a signal intensity close to the bistability threshold and show slower and noisier responses. The OFF to ON plots start with the systems at an OFF steady state (low levels of RRP) corresponding to a low value of k1 or a high value of k2. At time zero, there is an instantaneous increase in k1 or decrease in k2. The ON to OFF plots start with the systems at an ON steady state (high levels of RRP) corresponding to a high value of k1 or a low value of k2. At time zero, there is an instantaneous decrease in k1 or increase in k2. The values for k1 or k2 are chosen to be below, next to and above the threshold value at which the system switches from OFF to ON, or from ON to OFF. See text for further details.

The simulation experiments performed were similar to those described in experiments 1–4 of the previous section, although with a smaller number of data points. Figures 4 and 5 show the results of these simulations.

The OFF→ON plots start with the system at the OFF steady-state (low concentration of active RR) corresponding to a low value of k_1_ (k_1_ = 10^−5^ s^−1^) or a high value of k_2_ (k_2_ = 10 s^−1^), and depict the temporal trajectory of the RRP concentration after an instantaneous increase in k_1_ or decrease in k_2_, for three different values of k_1_ and k_2_.

The ON→OFF plots start with the system at the ON steady-state (high concentration of active response regulator) corresponding to a high value of the signal k_1_ (k_1_ = 10 s^−1^) or a low value of k_2_ (k_2_ = 5·10^−6^ s^−1^), and depict the temporal trajectory of the RRP concentration after an instantaneous decrease in k_1_ or increase in k_2_, for three different values of k_1_ and k_2_.

The simulation results for three different signal intensities are plotted in Figures 4 and 5. Three independent simulations are shown for each signal intensity. The values of k_1_ and k_2_ in each trajectory are chosen to be below, next to and above the threshold value at which the system switches from OFF to ON, or from ON to OFF (in the cases in which this threshold exists). Because each system has a different threshold value, the parameter scan is different for each plot.

The results from the analysis of the continuous model are consistent with the stochastic simulations: as discussed in the previous section (Figure 3), in systems with a signal range of bistability the response times increase when the signal intensity is near the threshold value at which the system exits the bistability region. One can see in Figures 4 and 5 that, in many cases, the curves that correspond to a signal that is just outside of the bistability range do not reach steady state during the simulation time. These curves correspond with the peaks in Figure 3.

Furthermore, our simulations predict that the systemic response becomes noisier as the signal intensity approaches the threshold value for bistability. Just above and just below this value there is an increase in the stochastic fluctuations of the system. This can be seen because the triplicate curves corresponding to these values in Figures 4 and 5 are much more different among themselves than the triplicate curves for the signals away from this threshold.

The response in the systems A, B and C is noisier when k_1_ is modulated than when k_2_ is modulated. The OFF to ON trajectories of Model B after an instantaneous decrease in k_2_ confirm that the turn OFF of this system due to an increase in k_2_ is irreversible and the system can't return to the ON state (see Figure 2 panel C). The system C does not have a bistability region in its k_2_-response curve (see Figure 2 panels E and F). Therefore, we don't find a range of k_2_ values for which the systemic response becomes slower and noisier.

### Robustness of the analysis

The analysis thus far was done using the specific set of parameter values reported in Table 2. In order to study the generality of the results we performed sensitivity analyses of the bistability to changes in the different parameter values and concentrations of the systems. The results of the controlled and uncontrolled comparison between Model A and Model B or C with respect to the effect of changing parameter values on a possible bistable response of the TCS are summarized in Table 3. The detailed results are shown in Figure S2, where we show a set of two-dimensional sections of the multidimensional parameter space in which bistability is observed.

**Table 2:** Basal values for the parameters and concentrations of the models in Figure 1.

Kinetic constant	Value
k_1_	e 0.1 s^−1^
k_2_	0.0005 s^−1^
k_3_	0.5 µM^−1^ s^−1^
k_4_	0.5 s^−1^
k_5_	1.5 s^−1^
k_6_	0.5 s^−1^
k_7_	0.05 µM^−1^ s^−1^
k_8_	0 s^−1^ (monofunctional SK)/f 0.05 s^−1^ (bifunctional SK)
k_9_	f0.5 s^−1^
k_10_	g0.5 µM^−1^ s^−1^
k_11_	0.5 µM^−1^ s^−1^
k_12_	0.5 s^−1^
k_13_	0.025 s^−1^
ak_14_	0.5 µM^−1^ s^−1^
k_15_	0.5 s^−1^
bk_16_	0.005 µM^−1^ s^−1^
ak_17_	0.5 µM^−1^ s^−1^
k_18_	0.5 s^−1^
**Proteins**	**Total Concentrations**
RR	6 µM
SK	0.17 µM
Ph	0.17 µM
cTC_SK_	1.17 µM
dTC_RR_	10 µM

a These values were chosen in such a way that the affinity of the TCS proteins with the third component would be similar to the affinity between the SK and the RR.

b The value for this parameter was chosen to be one order of magnitude larger than that representing SK autodephosphorylation, because the TC_SK_ enhances SK autodephosphorylation.

c TC _SK total_ is the total amount of the third component in Model B. This third component protein binds the SK of the TCS module. The amount for this protein was chosen taking into account that basal mRNA levels for RetS in GEO micro profiles of *Pseudomonas aeruginosa* are between 2 and 10 times higher than those of GacS. GacS is an SK and RetS is its cognate TC_SK_ [47].

d TC _RR total_ is the total amount of the third component in Model C. This third component protein binds the phosphorylated RR of the TCS module. The amount for this protein was chosen to be in the same order of magnitude as that of the RR, as is done in reference [43].

e This is the average value for the autophosphorylation catalytic constant between *Salmonella typhimurium* and *Escherichia coli* [*16*].

f It should be noted that, for Model C, this value for the phosphatase rate constant could be as high as 0.14 in *Escherichia coli* [*16*].

g Although some measurements have suggested that the affinity between non-phosphorylated forms of the SK and RR is much lower than the affinity between phosphorylated forms of the proteins [48], more recent measurements suggest the opposite [10].

**Table 3:** Percentage of parameter space where bistable responses are possible^a^.

	Model A	Model A|B	Model B	Model A|C	Model C
**Monofunctional**					
Input signal: change in k_1_	8	7.56	6.04	8.98	6.74
Input signal: change in k_2_	11.36	21.87	17.52	9.11	4.01
**Bifunctional**					
Input signal: change in k_1_	4.85	4.89	3.81	2.24	4.98
Input signal: change in k_2_	11.44	7.77	4.11	1.84	4.31

a Some bidimensional sections of the multidimensional parameter space of bistability are shown in Figure S2. The results show that in TCS with a bifunctional SK, both a TC_SK_ and a TC_RR_ cause a decrease in the size of the parametric region of bistability, with one exception: Model C has a larger parametric region of bistability when the signaling target is SK autophosphorylation (k_1_). However, in systems with a monofunctional SK, a TCSK causes an increase and a TCRR causes a decrease in the size of the parametric region of bistability if the environment modulates the SK dephosphorylation (k_2_). A|B stands for Model A controlled for Model B. A|C stands for Model A controlled for Model C.

Overall, a system with a TC_SK_ appears to have a wider parameter range of bistability if the SK is monofunctional, and a lower parameter range of bistability if the SK is bifunctional, while a system with a TC_RR_ appears to have a lower parameter range of bistability, for systems with either a monofunctional or a bifunctional SK, when either system is compared to a prototypical TCS. However, if the comparison between Model A and Model B or C is controlled, then we see that the robustness of the parameter range of bistability is larger in the prototypical TCS (Model A) with only one exception: in systems with a bifunctional SK, Model C has a more robust parameter range of bistability.

### Effect of changes in SK-independent RRP dephosphorylation and SKRR affinity on bistability

SK-independent RRP dephosphorylation and SKRR complex formation are needed for bistable responses to exist in Models A, B, and C. In order to investigate how quantitatively changing these features affects bistability we performed the following computational experiments (Table 4). We independently and simultaneously changed the values for k_8_ (the reaction that regulates dephosphorylation by the SK) and k_9_ (changing the rate of dissociation between SK and RR) between 10^−6^ and 10. Then, we calculated the steady state(s) for each system at different values of the signal represented by the parameters k_1_ or k_2_. k_1_ and k_2_ were independently and systematically scanned between 10^−6^ and 10 in logarithmic space at intervals of 0.01 units. The results are shown in Table 5 and Figure S3. Table 3 shows that, overall, bistability is possible in Model C in a smaller interval of parameter values than that for Models A and B. However, the picture changes when we analyze only the parameters that directly influence the necessary conditions for bistability (k_8_, k_9_, k_10_). For these parameters, Model C is the system where overall bistability is possible in a wider range of parameter values, followed by Model B. Model A is the one where bistability is limited to a smaller region of parameter values. Nevertheless, when Model A is controlled to have signal-response curves that are as similar as possible to those of either Model B or Model C, Model A becomes the system where bistable responses can occur in a larger fraction of the space for k_8_, k_9_, and k_10_. For values of k_8_ below a threshold that depends on the system and is lower in Model B than in Model A, bistability is present in both models. Within the range of k_8_ values that permit bistability, an increase in k_8_ causes an increase in the k_2_ range of bistability (up to approximately six orders of magnitude for k_2_ at the threshold value for k_8_). This is so, despite the enlargement of the fraction of RRP dephosphorylated by SK, because the increase in k_8_ causes an increase in the concentration of the SKRR dead-end complex (see Figure S4). As k_8_ decreases, the range of signal k_2_ in which the models show bistability decreases steadily for a few orders of magnitude. Then, a lower boundary is reached and bistability is observed for one or less than one order of magnitude of k_2_ signal, independently of the value for k_8_.

**Table 4:** Experiments to analyze the effect of changes in different parameter values and protein concentrations on the range of bistability for the alternative TCS modules^a^.

Sensitivity to changes in	Parameter	Range of scanning	Parameter	Range of scanning
**Formation of the SKRR dead end complex**	k_9_	10^−6^–10 s^−1^	k_10_	10^−6^–10 µM ^−1^ s^−1^
**Ratio between SK_total_ and RR_total_.**	SK_total_	10^−3^–10^3^ µM	RR_total_	10^−3^–10^3^ µM
**Ratio between SK_total_ and TC_SK total_.**	TC_SK total_	10^−3^–10^3^ µM	SK_total_	10^−3^–10^3^ µM
**Ratio between RR_total_ and TC_RR total_.**	RR_total_	10^−3^–10^3^ µM	TC_RR total_	10^−3^–10^3^ µM
**Formation of the SKRR dead-end complex and rate of RRP dephoshoprylation by SK**	k_8_	10^−6^–10	k_9_	10^−6^–10 s^−1^

a The steady state(s) for the three models by scanning a)k_1_ (SK autophosphorylation reaction rate constant) and b)k_2_ (SKP autodephosphorylation reaction rate constant) between 10^−6^ and 10 at different values of the parameters named in the table (see text for details).

**Table 5:** Percentage of parameter space where a bistable response is possible for Models A, B, and C^a^.

	Experiment	Model A^b^	Model A|B^c^	Model B^b^	Model A|C^c^	Model C^b^
**Bifunctional**						
	k_8_,k_9_,k_2_	1.8	5.3	2.5	17.8	8.1
	k_9_,k_10_,k_2_	1.2	0.5	2.7	5.7	4.3
	SKt,RRt,k_2_	0.6	NA	1.4	NA	1
	SKt,TCt,k_2_	NA	NA	10.9	NA	3
	k_8_,k_9_,k_1_	35.5	33.4	36.7	47.9	39
	k_9_,k_10_,k_1_	11.3	10.5	11.9	14.3	13.9
	SKt,RRt,k_1_	14.1	NA	16	NA	14
	SK,TCt,k_1_	NA	NA	31.3	NA	26.4
**Monofunctional**						
	k_9_,k_10_,k_2_	11.9	8.2	15.6	20.9	13.1
	SKt,RRt,k_2_	7.7	NA	9.2	NA	6.2
	SKt,TCt,k_2_	NA	NA	4.4	NA	10
	k_9_,k_10_,k_1_	41.4	40.1	42.7	49.3	40.9
	SKt,RRt,k_1_	31.2	NA	34	NA	27.9
	SK,TCt,k_1_	NA	NA	75.3	NA	30.7

a A|B stands for Model A controlled for Model B. A|C stands for Model A controlled for Model C.

k_i_: kinetic constants for the reactions in the systems shown in Figure 1. SKt: total concentration of SK. RRt: total concentration of RR. TCt: total concentration of third component protein. The parameter space for k_i_,k_j_, and k_k_ was scanned between absolute values of 10^−6^ and 10 for each of the parameters. Sampling was uniform in logarithmic space.

b Percentage of the parameter space of k_i_, k_j_ and k_k_ where bistability is found for Models A, B, and C respectively.

c Percentage of the parameter space where bistability is found in Model A controlled for B and for C, respectively.

NA Non Applicable. Mono functional systems have k_8_ = 0. The concentration of TC = 0 in Model A. Model A can not be scanned with respect to the concentration of SK in the controlled comparisons, because SK is independently fixed to make the dynamical response of Model A more similar to those of Models B and C.

Given that the formation of a dead-end complex between SK and RR is a necessary condition for bistability, we also want to understand the isolated effect of different fractions of RR and SK being sequestered into this complex on bistability. To understand the effect of changing the amount of SKRR dead-end complex on the signaling range in which the systems can be bistable we performed the following numerical experiment. First, we took each model from Figure 1. Then, we systematically scanned the values of the parameters k_9_ and k_10_, independently and simultaneously, between 10^−6^ and 10 in logarithmic space at intervals of 0.01 units. These parameters regulate the amount of SKRR that is formed. Finally, for each pair of values for k_9_ and k_10_, we independently calculated the steady state(s) for each system at different values of the signal represented by k_1_ or k_2_. Each of these parameters was independently and systematically scanned between 10^−6^ and 10 in logarithmic space at intervals of 0.01 units. The results are shown in Table 5 and Figure S3.

Bistability can be found only for intermediate steady state concentrations of SKRR. If too little or too much SKRR is formed, then no bistable response is possible. Overall, for bifunctional TCS, Model C has the largest range of SKRR steady state concentrations for which bistability is possible, followed by Model B. In its uncontrolled form Model A has the smallest interval of SKRR steady state concentrations where bistability is permitted. This interval of concentrations decreases further when Model A is controlled to be comparable to Model B. However, when Model A is controlled to be comparable to Model C, the range of SKRR steady state concentrations that enable bistability becomes the largest of the three systems. In monofunctional TCS, Model C has a smaller range of SKRR steady state concentrations for which bistability is possible than Model B.

The notion that Model C is the one in which bistable responses are less sensitive to changes in the steady state concentrations of SKRR (in consequence of changing the affinity between SK and RR) is misleading. Bistability is only found in this model if the affinity between the dephosphorylated forms of SK and RR is much larger than that between SKP and RR or SK and RRP. Given that the affinity between all forms of SK and RR was measured as similar, it is not likely that bistability can be found *in vivo* in systems that are represented by this model.

A similar experiment was made by changing independently and simultaneously the total amount of SK and RR, followed by independent calculation of the steady state(s) for each system at different values of the signal represented by k_1_ or k_2_. Again, each of the parameters was independently and systematically scanned between 10^−6^ and 10 in logarithmic space at intervals of 0.01 units. The results are shown in Table 5 and Figure S3. They are consistent with the situation described for changes in k_9_ and k_10_.

### Effect of the SK/TC_SK_ and RR/TC_RR_ concentration ratios on bistability

In order to understand how the relationship between the total amounts of SK (RR) and TC_SK_ (TC_RR_) influences the signaling range in which bistable responses are possible, we have performed a number of computational experiments. First, we took Models B and C from Figure 1. Then, we systematically, simultaneously and independently scanned the total amounts of SK (RR) and TC_SK_ (TC_RR_) in Model B (Model C), as described in Table 4. Finally, for each total amount of SK (RR) and TC_SK_ (TC_RR_), we calculated the steady state(s) for each system at different values of the signal represented by k_2_. This parameter was also systematically scanned between 10^−6^ and 10 in logarithmic space at intervals of 0.01 units. The results are shown in Figure S3. We also performed similar test replacing k_2_ by k_1_.

The range of signal k_2_ for which Model B can show a bistable response is observed to be dependent on the TC. Bistability is observed only within a narrow band of the SK-TC_SK_ concentration space. Outside of this band, a bistable response cannot be observed. The range of total amount of SK in the system that may lead to a bistable response remains approximately constant for low total amounts of TC_SK_. However, within the band of total SK and TC_SK_ in which bistability is observed, as total TC_SK_ increases, the range of total SK amount that can generate bistable responses also increases. At concentrations of TC_SK_ between approximately 2 and 7 µM, we find bistability for total SK concentrations between 0.2 and 0.001 µM or lower. At higher total TC_SK_ concentrations, only small amounts of SK are available in free form. This prevents formation of the SKRR dead-end complex that is required for bistability.

As is the case in Model B, bistability in Model C can be achieved in a narrow band of the concentration space. However, within the range of values of this simulation, whatever the concentration of TC_RR_, the system can always show bistability.

## Discussion

### Summary of the comparisons

Tables 6 and 7 summarize our findings regarding the different physiological criteria that are relevant for TCS signal transduction and can be asserted from the analysis of our models. In general, if the signaling target is SK autophosphorylation Model C responds at lower signaling intensities, followed by Model A, and finally by Model B. If the signal enhances SK dephosphorylation, Model B is the one that responds at lower signal intensities, followed by Model A, and Model C. This causes Model C to be in an ON state for a wider signaling range, and Model B to be in an ON state for a narrower signaling range, in comparison with Model A.

**Table 6:** Summary of the comparison of physiologically relevant criteria between the alternative designs for monofunctional TCS^a^.

		MONOFUNCTIONAL				
Signaling target	Physiological criterion	Model A	Model B	Model C	Model A|B	Model A|C
**Phosphorylation of SK (k_1_)**	Sensitivity to signal	+++	++	+++++	++	++++
	Signaling range of bistability	+++	++	+	++	++++
	Fraction of parameter space with bistability	++++	+	++	+++	+++++
	Noisy response	+++	+++++	+	++++	++
	Fast OFF→ON response time	++++	++	+++	+	+++++
	Fast ON→OFF response time	+++	+	++++	++	+++++

		Model A	Model B	Model C	Model A|B	Model A|C
**Dephosphorylation of SKP (k_2_)**	Sensitivity to signal	+++	+++++	++	++++	++
	Signaling range of bistability	++	++++	−	++++	−
	Fraction of parameter space with bistability	+++	++++	+	+++++	++
	Noisy response	+	+	+	+	+
	Fast OFF→ON response time	+++	++++	+	+++++	++
	Fast ON→OFF response time	+++	++++	+	+++++	++

a The model with the largest number of “+” signs for a given criterion is the one with the best performance with respect to that criterion.

A|B stands for Model A controlled for Model B. A|C stands for Model A controlled for Model C.

**Table 7:** Summary of the comparison of physiologically relevant criteria between the alternative designs for TCS with bifunctional SK^a^.

		BIFUNCTIONAL				
Signaling target	Physiological criterion	Model A	Model B	Model C	Model A|B	Model A|C
**Phosphorylation of SK (k_1_)**	Sensitivity to signal	++	+	++++	+	+++
	Signaling range of bistability	+++	++	+	++	−
	Fraction of parameter space with bistability	+++	++	+++++	++++	+
	Noisy response	+++	+++++	++	++++	+
	Fast OFF→ON response time	++++	++	+++	+	+++++
	Fast On→OFF response time	+++	+	++++	++	+++++

		Model A	Model B	Model C	Model A|B	Model A|C
**Dephosphorylation of SKP (k_2_)**	Sensitivity to signal	++++	+	++	+	+++
	Signaling range of bistability	+++	−	−	−	−
	Fraction of parameter space with bistability	+++++	++	+++	++++	+
	Noisy response	+	+	+	+	+
	Fast OFF→ON response time	+++	++++	+	+++++	++
	Fast ON→OFF response time	++	+++	+	++++	+++++

a The model with the largest number of “+” signs for a given criterion is the one with the best performance with respect to that criterion.

A|B stands for Model A controlled for Model B. A|C stands for Model A controlled for Model C.

The system with the largest range of signaling in which it can show a bistable response depends on both, the type of SK in the module and the SK activity (autophosphorylation or autodephosphorylation) that is targeted by the signal. For TCS with monofunctional SK, Model A has the largest signaling range for bistability, as well as the largest fraction of parameter space where such bistability can be observed, if the environment modulates SK phosphorylation. In contrast, Model B has the largest signaling range for bistability, as well as the largest fraction of parameter space where such bistability can be observed, if the environment modulates SK dephosphorylation. For TCS with bifunctional SK, Model B has the largest signaling range for bistability if the environment modulates SK phosphorylation. However, it is Model C that has the largest fraction of parameter space where bistability can be observed. In contrast, Model A has the largest signaling range for bistability, as well as the largest fraction of parameter space where such bistability can be observed, if the environment modulates SK dephosphorylation.

Modulation of SK dephosphorylation leads to responses that have an equally small amount of noise in all Models. However, modulation of SK phosphorylation leads to noisier responses in Model B, followed by Model A and finally Model C.

As is the case with bistability, the model with fastest response times depends on the type of SK in the module and on the SK activity (autophosphorylation or autodephosphorylation) that is targeted by the signal. Both in systems with monofunctional and bifunctional SK, Model A is the fastest to respond (Model C is the slowest) whether the signaling target is the autophosphorylation or the autodephosphorylation of the SK, with only one exception: Model B turns ON faster if SK autophosphorylation is modulated directly. The response times of Models A and B are similar, but Model C tends to be much slower than Model A.

### Biological Relevance

Bacteria often sense and adapt to changes in the environment through TCS and phosphorelays. A question that this work addresses is how variations to the prototypical TCS by means of an accessory third protein that either binds the SK or the RR affect the dynamical behavior of the TCS module.

TCS can, in principle, mediate both gradual and switch like (bistable) responses to environmental stimuli [32], [33]. The switch-like response has typically been associated to the positive feedback introduced by genetic regulatory loops in the regulation of autogenous TCS. Nevertheless, such feedback does not necessarily imply the existence of bistability [34]. In fact, genetic positive feedback loops are not strictly necessary for the existence of bistable responses in prototypical TCS. Such responses can also come about through post-translational regulation of bacterial signal transduction networks [25], [35]. Namely, bistability is possible in prototypical TCS if a reversible dead-end complex is formed between the dephosphorylated SK and RR and if a sufficient amount of RRP is dephosphorylated independently of the SK phosphatase activity [25].

TC proteins that regulate signal transmission to prototypical TCS have been known for years [36], [37]. However, only recently have such interactions been proposed as a way to integrate non-cognate signals in the TCS regulated responses. In fact, these interactions have been reported in TCS that are responsible for regulating both, resistance to antibiotics and virulence [6], [7], [8], [9], [12], [13], [14], [15].

Biological examples of the first situation can be found in the PmrB/PmrA/PmrD system. The third component PmrD binds and stabilizes the active form of the RR, PmrA. This system regulates antibiotic resistance in *Salmonella* and other bacteria. Various studies of the PmrA/PmrB/PmrD system suggest that this TC_RR_ could be an intermediate evolutionary step to evolve indirect regulation of the TCS [12], [16], [17], [22], [38]. The feedforward connector loop formed by PmrD is presented as a design that speeds up activation and slows deactivation of the gene expression of the proteins in the TCS [17]. Our results suggest that this may not be so in non-autogenous TCS. If the TCS has a TC_RR_, loss of this protein will make the corresponding prototypical TCS faster to turn ON and OFF (Table S1 and Figure S1). In fact, if the steady state response curve of the prototypical TCS is mathematically controlled to be as similar to that of the TCS with a TC_RR_ as possible, then that prototypical system is always faster. A TC_RR_ appears also to be a feature that decreases the fraction of parameter space in which bistable responses are possible (Tables 3 and 5), except in TCS with a bifunctional SK and when the environment modulates SK autophosphorylation. Thus, a TC_RR_ creates a TCS module that is less likely to show bistable responses and slower in responding to environmental signals, which it can sense at lower intensities than the prototypical TCS without any TC, if SK phosphorylation is modulated.

Antibiotic resistance is arguably a trait whose response should be gradual and proportional to the amount of antibiotic found by the bacteria to increase its survival chances. If this is not so, and a bistable response is possible, bacteria can be made more sensitive to antibiotics [39] and therefore their survival will be hindered. Given that bistability has been observed in the antibiotic resistance of some bacteria [39], a TC that binds the RR would reduce the possibility of such bistable response, potentiating adaptation and tolerance to threatening stress challenges. In addition, having such a TC could enable a response at low antibiotic concentrations, thus increasing the chances of survival for the organism.

The other well studied example of a TC interacting with the TCS is the RetS/GacS/GacA system, where RetS reversibly binds and inactivates the SK GacS. This system regulates virulence in *Pseudomonas aeruginosa*. Recently, it has been shown that the GacS/GacA TCS acts exclusively through the regulation of the transcription of two genes, *rsmY* and *rsmZ* [40]. The product of these genes are two untranslated small regulatory RNAs (sRNAs), RsmY and RsmZ, that counter translational repression exerted by the RNA-binding protein RsmA on target mRNAs encoding virulence factors. There is an additional SK, LadS, that appears to counter the action of RetS on GacS. However, this effect is indirect, as not direct physical interaction between GacS and LadS was observed [10]. It may be that LadS sequesters RetS, as RetS does with GacS. Our analysis of a TCS with a TC_SK_ reveals that this module will respond at signal intensities that are slightly higher (lower) than those of the prototypical TCS, if SK authophosphorylation (autodephosphorylation) is directly modulated. Furthermore, if one is to synthetically change a TCS module and create an artificial circuit with a TC_SK_, the engineered circuit will typically respond faster to signals if the environment modulates SK dephosphorylation. However, evolution can eventually equalize response times by changing the SK concentration of the module and making both TCS modules have steady state response curves that are similar. A TC_SK_ can increase the signaling range in which a bistable response is possible (Table 5). Bistability could be advantageous when the system has to choose between two different operational states [35], [41], as is often the case for virulent organisms. For example, *Mycobacterium tuberculosis* is a persistent organism in the lungs of 2/7 of the world population [42]. However, only under certain conditions that are not yet completely clear does this organism causes tuberculosis [42]. Bistability could provide populations with the capacity to sample which type of phenotype is more advantageous at different times and enhance survival of the organisms through bet-hedging strategies [43], [44].

Experiments to test the existence of bistability in a TCS with a TC_SK_ could be as follows, taking the RetS/GaS/GacA system as an example. First, determine if the system can show bistable response: incubate two *Pseudomonas aeruginosa* strains (a wild type strain, with the TC protein RetS, and a RetS mutant strain, without the TC protein) at different environmental conditions of inducing signal intensity, allow the cells to approach a steady state and measure the levels of expression of the sRNAs RsmY and RsmZ. In a TCS module with a monostable gradual response, the level of expression of the output molecules should be proportional to the environmental inducing signal intensity: at intermediate signal intensities there is an intermediate amount of output molecule. However, if the RetS/ GacS/GacA response is bistable, we will find that, for intermediate intensities of inducing signal, the measured levels of RsmY and RsmZ in single cells are distributed in a bimodal manner, with low and high levels (but no intermediate levels) of this sRNAs. If bistability is present and the *in vivo* effect of RetS is to amplify the signaling range for which a bistable response is possible, we will find that this bimodal distribution of the measured levels of RsmY and RsmZ in single cells of the RetS mutant strain will be observed in a smaller range of signal values. To investigate if the results of our simulations are valid for *in vivo* conditions and if the RetS/GacS/GacA system could have an irreversible response (as observed in Figure 2 C), we can incubate both strains in a high-stimulus environment, allow the cells to approach a steady state and measure the levels of the sRNAs RsmY and RsmZ . If the in vivo system behaves as its *in silico* proxy, when the stimulus is removed (transfer the cells to a non-inducing environment), we will find that in RetS mutant cells the levels of RsmY and RsmZ shift from a low value to a high value, but in wild type cells the levels of RsmY and RsmZ remain at a low value.

The arguments discussed thus far explain part of the biological relevance of our work. Another interesting aspect of it regards the modulation of SK autophosphorylation and dephosphorylation. Currently the community is inclined to assume that dephosphorylation is the target of modulation by environmental signals in many cases. However, to our knowledge, conclusive experiments that decide the issue are still lacking in most systems and it is still unclear whether the physiological signal modulates SK autophosphorylation (k_1_) or SKP dephosphorylation (k_2_). That is why we have performed our simulations taking as a signal both changes in k_1_ and k_2_. An unexpected result of our simulations may shed some light on this issue, and allow us to hypothesize which one of the reaction rates is modulated by the signal in the case of TCS with a TC. We have found that, for TCS with a bifunctional SK, a TC decreases the possibility of a bistable response. For TCS with a monofunctional SK, the same effect is observed if the signal modulates k_1_. However, if the signal modulates k_2_, a TC_SK_ increases the range of signal intensities in which a TCS can show bistability, and a TC_RR_ decreases it. Thus, for TCS with a monofunctional SK, the results suggest that the physiological signal should modulate SK dephosphorylation (k_2_) both when bistability is an advantageous feature in the function of a TCS with a TC_SK_ component, and when bistability is a disadvantageous feature in the function of a TCS with a TC_RR_. Conversely, the physiological signal should modulate SK autophosphorylation (k_1_) when bistability is a disadvantageous feature in the function of a TCS with a TC_SK_.

The work presented in this paper provides motivation for further analyses of the TCS responsible for regulating virulence and antibiotic resistance, providing clues as to possible mechanisms to both decrease virulence and antibiotic resistance. In the case of virulence, whenever it is regulated by a TCS of the type analyzed here, simultaneously targeting the TC and the SK appropriately could prevent the organism from becoming virulent. In the case of antibiotic resistance, targeting the TC and its interaction with the RR could be used to facilitate locking the bacteria in an antibiotic-sensitive state and facilitate treatment of infections.

## Methods

### Equations

In order to compare the physiological behavior of the three systems in Figure 1, we must create a mathematical representation for each of the networks. The positive and negative terms of each ODE correspond to individual reactions that give rise to the synthesis and degradation of the reactant, respectively. Each reaction is considered to be mass action.

Because the turnover times for protein synthesis and degradation are much higher than those for the phosphorylation-dephosphorylation reactions, we consider the total amount of each participating protein to be approximately constant. Thus,
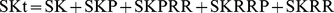


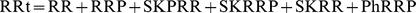


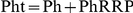


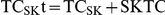


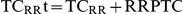
where SKt, RRt, Pht, TC_SK_t and TC_RR_t are constant and denote the total amount of SK, RR, Ph, TC_SK_ and TC_RR_ respectively.

Applying all simplifications, the differential equations for Model A become:
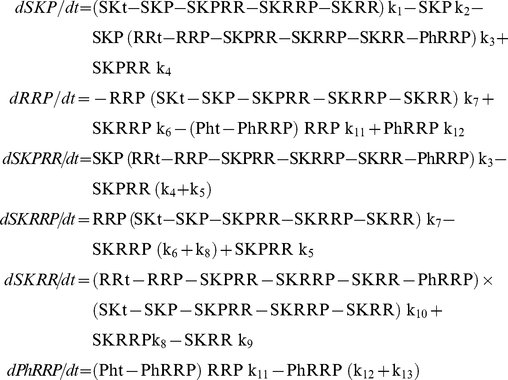



Applying all simplifications, the differential equations for Model B become:
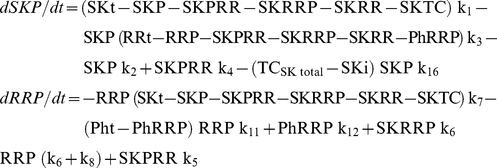


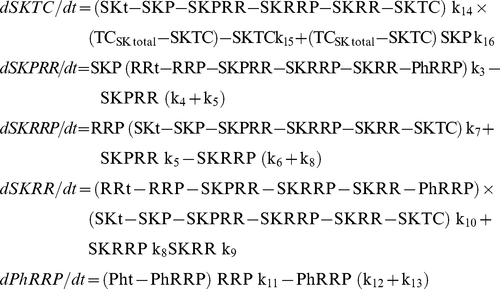



Applying all simplifications, the differential equations for Model C become:
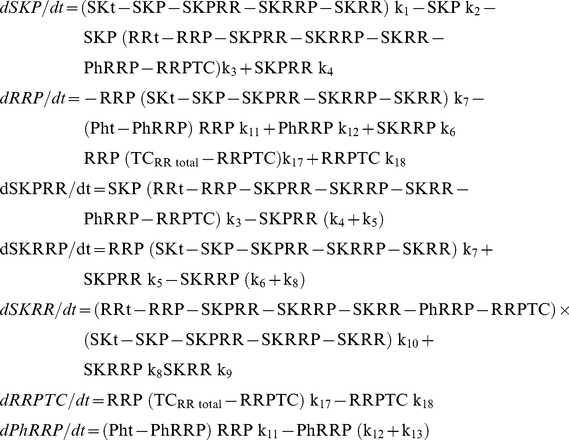
The parameters for the models are given in Table 1. All these parameters have an experimental basis, clearly presented in Igoshin *et al.* [25].

### Mathematically controlled comparisons

We aim at comparing the physiological behavior of the three models in order to understand if the presence of a TC in a TCS module causes intrinsic differences to the potential physiological responses that the modules can have. To make sure that the differences observed in the behavior of the systems that are being compared are due to the presence of the TC, the comparisons must be made in a controlled way. For this we use the method of mathematically controlled comparisons [31]. This method requires that all components and processes that are common to the alternative models that are to be compared are made numerically equal, making the models internally equivalent. In contrast, the components and processes that are different between the alternative models are degrees of freedom that nature could potentially use to compensate the changes in the physiological responses caused by the differences between systems. In this case, the systems with a TC invest additional resources to synthesize a new protein that binds either the SK or the RR and modulates their phosphorylation state. All new processes of Models B and C with respect to Model A are due to the presence of this TC. In order to control the comparison between TCS with TC and the prototypical TCS, the prototypical system (Model A) should also be allowed to invest additional resources in adjusting the total amount of the SK or the RR. These adjustments will allow the prototypical system to have a physiological response that is as similar as possible to that of the model with a SK-binding or a RR-binding TC (Models B and C, respectively). This control condition ensures maximal external equivalency between the models. Once the maximum equivalency is achieved between the compared models, the remaining behavioral differences can be related to the presence of the TC.

To determine the changes in the total amount of SK or RR that make the physiological responses between Model A and Models B or C as similar as possible, we have used a minimum square differences method. We have calculated the steady state responses of the system in Models B and C to changes in the input phosphorylation or dephosphorylation rate of the modules, by calculating the steady state concentration of RRP in Models B and C, at input signal strengths between 10^−6^ and 10. These curves were then used individually to fit Model A and calculate the concentration of SK and/or RR that would minimize the differences in the steady state RRP concentration between Model A and Models B or C, independently. All calculations were done using Mathematica. The best fits are achieved by allowing the total amount of SK to change in Model A. The values for the total amount of SK in Model A that minimize the differences between the responses of this model and Model B or Model C are shown in Table 8.

**Table 8:** Values of SK_total_ in Model A used in the mathematically controlled comparisons ^a^.

[SK_total_] in Model A (µM)				
	Monofunctional		Bifunctional	
	k_1_	k_2_	k_1_	k_2_
**Model A|B**	0.13	0.13	0.14	0.14
**Model A|C**	0.50	0.50	0.90	0.90

These values are chosen to make the signal-response curves of the prototypical TCS (Model A) and the system with a third component (Models B or C) as similar as possible, for responses to an environmental stimulus that modulates either k_1_ (SK autophosphrylation kinetic constant) or k_2_ (SKP autodephosphrylation kinetic constant). A|B stands for Model A controlled for Model B. A|C stands for Model A controlled for Model C.

### Calculations

All simulations were performed in Mathematica [45] and COPASI [46]. Analyses of regions of bistability were done in Mathematica, using in-house scripts.

## Supporting Information

Figure S1 **Temporal responsiveness curves of Models A, B, and C.** The systems are at an initial steady state and, at time zero, the signal, represented in the x axis, changes instantaneously and the time it takes for the system to get to within 90% of the new steady state is measured and plotted in the y axis. A–D: Response times of TCS with monofunctional SK. E–H: Response times of TCS with bifunctional SK. The OFF to ON plots start with the systems at an OFF steady state (low levels of RRP) corresponding to a low value of k_1_ (A, C, E, G) or a high value of k_2_ (B, D, F, H). The signal is then changed to increase the steady state level of RRP. The ON to OFF plots start with the systems at an ON steady state (high levels of RRP) corresponding to a high value of k_1_ or a low value of k_2_. The signal is then changed to decrease the steady state level of RRP. Peaks that indicate slower response times are located immediately outside the range of bistability. The lack of a peak in a curve can be due to monostability or irreversibility Absence of a dashed line indicates irreversible turning ON or OFF of the system (Model B in panel C ) or absence of bistability (see the signal-response curves of Figure 2). The difference between this Figure and Figure 3 is that the time curves for Model A are calculated with the total concentration of SK being the same in the three Models. The overall response times (equivalent to the sum of all the transient response times for each curve) is shown in Table S1.
(TIF)

Figure S2 **Effect of changing the parameter values on the range of bistability in the three TCS modules.** In the panels, the x-axis represents values for k_1_ (SK autophosphorylation rate constant) or k_2_ (SK dephosphorylation rate constant), and the y-axis represents values for each of the other reaction rate constants that are common to the three models (from k_2_ to k_13_). The region where bistability is possible is shaded in blue. The number above each set of plots represents the summation of all areas of bistability in a given model, that is, is a measure of the size of the parametric space of bistability. A, B: Comparison between Models A and B, with a monofunctional SK. C, D: Comparison between Models A and B, with a bifunctional SK. E, F: Comparison between Models A and C, with a monofunctional SK. G, H: Comparison between Models A and C, with a bifunctional SK.
(TIF)

Figure S3 **Percentage of parameter space where a bistable response is possible for Models A, B, and C.** Experiments as described in Table 4. The x and y axis represent the values of the scanned parameters, while the z-axis represents the orders of magnitude of signal for which there is a bistable response. The red projection represents the area of parameter space where bistable responses are possible. A – Bifunctional system, signal modulating dephosphorylation of the SK.; B – Bifunctional system, signal modulating the phosphorylation of the SK; C – Monofunctional system, signal modulating dephosphorylation of the SK.; D – Monofunctional system, signal modulating the phosphorylation of the SK. See text for details and discussion.
(TIF)

Figure S4 **Influence of the k_8_ value (SK bifunctionality rate constant) on the k_2_ range of bistability.** Within a k_8_ range of values, an increase in k_8_ causes an increase in the k_2_ range of bistability (panel a and b). This is so, despite an enlargement of the fraction of RRP dephosphorylated by SK (panel c), because of an increase in the SKRR concentration due to a higher value of k_8_ (panel d). The simulations were performed using the system represented by Model A.
(TIF)

Table S1 **Overall response times for the three systems modeled (uncontrolled comparison) ^a^.** ^a^ Results of the integral for the signal-response time function of Models A (uncontrolled), B and C. These values represent the area below each curve in Supplementary Figure 2, that is, the sum of the transient times for each response.
(DOC)
