# Supplementary figures and images for: Two Component Systems: Physiological Effect of a Third Component
Source: PLoS One. 2012 Feb 17;7(2):e31095. doi: 10.1371/journal.pone.0031095 (PMC3281920; doi:10.1371/journal.pone.0031095)

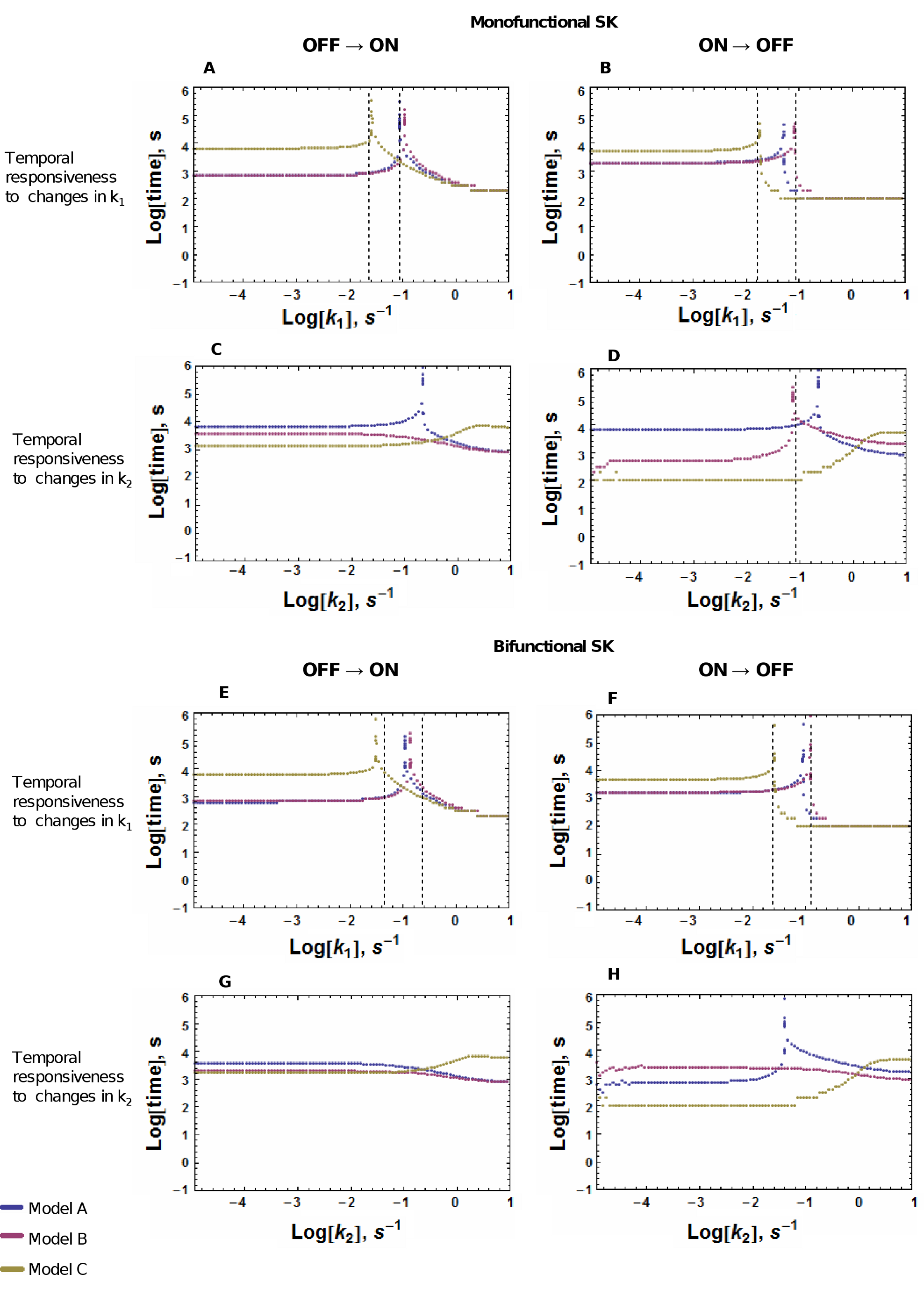

Supplement: Figure S1 — Temporal responsiveness curves of Models A, B, and C. The systems are at an initial steady state and, at time zero, the signal, represented in the x axis, changes instantaneously and the time it takes for the system to get to within 90% of the new steady state is measured and plotted in the y axis. A–D: Response times of TCS with monofunctional SK. E–H: Response times of TCS with bifunctional SK. The OFF to ON plots start with the systems at an OFF steady state (low levels of RRP) corresponding to a low value of k1 (A, C, E, G) or a high value of k2 (B, D, F, H). The signal is then changed to increase the steady state level of RRP. The ON to OFF plots start with the systems at an ON steady state (high levels of RRP) corresponding to a high value of k1 or a low value of k2. The signal is then changed to decrease the steady state level of RRP. Peaks that indicate slower response times are located immediately outside the range of bistability. The lack of a peak in a curve can be due to monostability or irreversibility Absence of a dashed line indicates irreversible turning ON or OFF of the system (Model B in panel C ) or absence of bistability (see the signal-response curves of Figure 2). The difference between this Figure and Figure 3 is that the time curves for Model A are calculated with the total concentration of SK being the same in the three Models. The overall response times (equivalent to the sum of all the transient response times for each curve) is shown in Table S1. (TIF) [file pone.0031095.s001.tif]

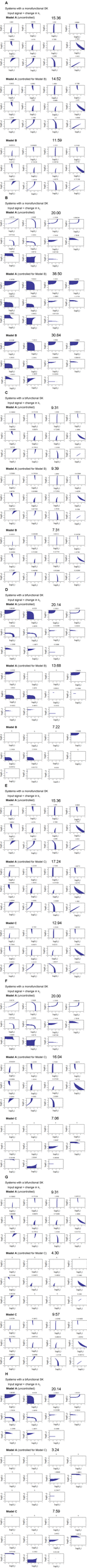

Supplement: Figure S2 — Effect of changing the parameter values on the range of bistability in the three TCS modules. In the panels, the x-axis represents values for k1 (SK autophosphorylation rate constant) or k2 (SK dephosphorylation rate constant), and the y-axis represents values for each of the other reaction rate constants that are common to the three models (from k2 to k13). The region where bistability is possible is shaded in blue. The number above each set of plots represents the summation of all areas of bistability in a given model, that is, is a measure of the size of the parametric space of bistability. A, B: Comparison between Models A and B, with a monofunctional SK. C, D: Comparison between Models A and B, with a bifunctional SK. E, F: Comparison between Models A and C, with a monofunctional SK. G, H: Comparison between Models A and C, with a bifunctional SK. (TIF) [file pone.0031095.s002.tif]

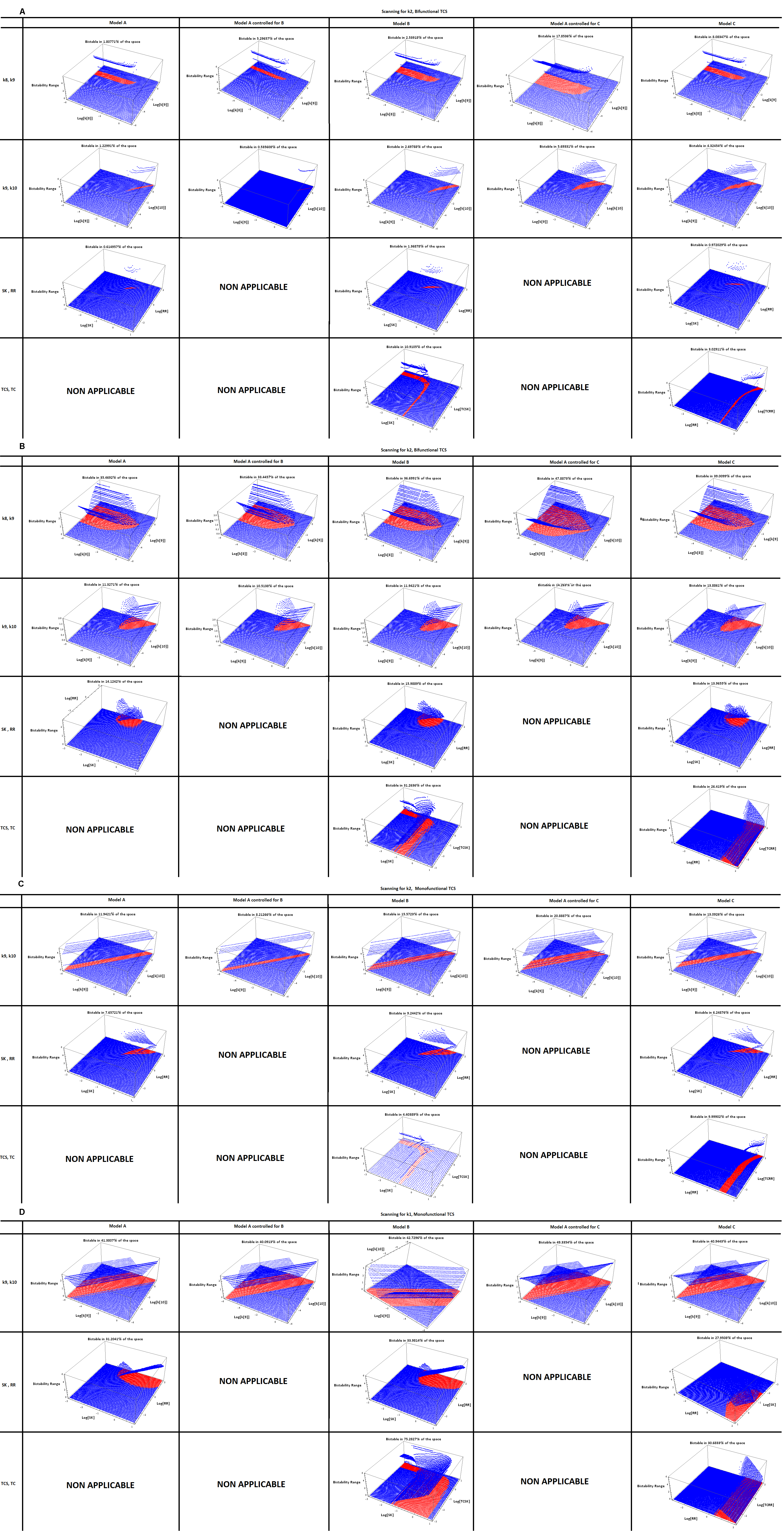

Supplement: Figure S3 — Percentage of parameter space where a bistable response is possible for Models A, B, and C. Experiments as described in Table 4. The x and y axis represent the values of the scanned parameters, while the z-axis represents the orders of magnitude of signal for which there is a bistable response. The red projection represents the area of parameter space where bistable responses are possible. A – Bifunctional system, signal modulating dephosphorylation of the SK.; B – Bifunctional system, signal modulating the phosphorylation of the SK; C – Monofunctional system, signal modulating dephosphorylation of the SK.; D – Monofunctional system, signal modulating the phosphorylation of the SK. See text for details and discussion. (TIF) [file pone.0031095.s003.tif]

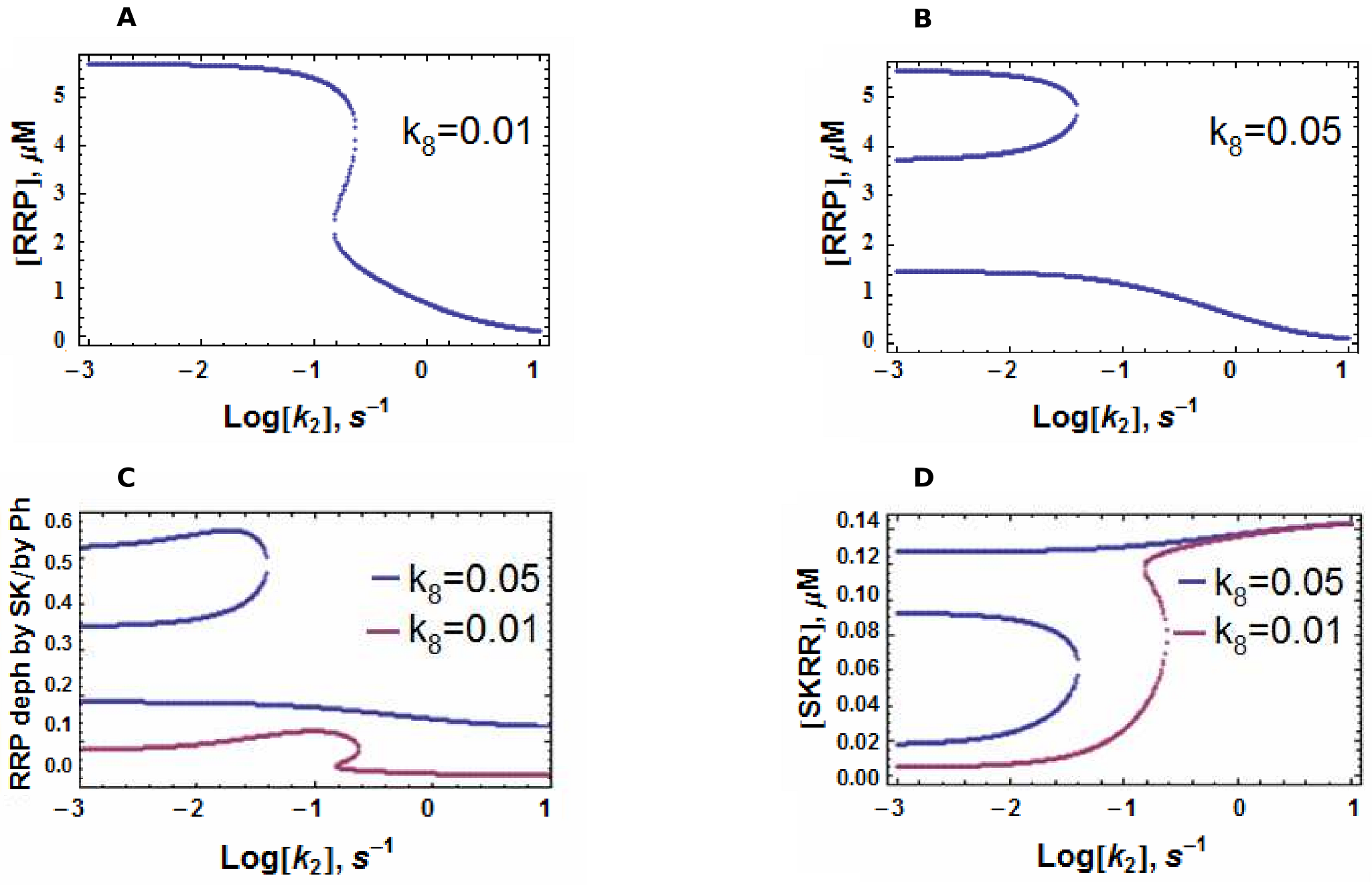

Supplement: Figure S4 — Influence of the k8 value (SK bifunctionality rate constant) on the k2 range of bistability. Within a k8 range of values, an increase in k8 causes an increase in the k2 range of bistability (panel a and b). This is so, despite an enlargement of the fraction of RRP dephosphorylated by SK (panel c), because of an increase in the SKRR concentration due to a higher value of k8 (panel d). The simulations were performed using the system represented by Model A. (TIF) [file pone.0031095.s004.tif]
