# Supplementary material for: Two Component Systems: Physiological Effect of a Third Component
Source: PLoS One. 2012 Feb 17;7(2):e31095. doi: 10.1371/journal.pone.0031095 (PMC3281920; doi:10.1371/journal.pone.0031095)
Supplement: Table S1 — Overall response times for the three systems modeled (uncontrolled comparison) a. a Results of the integral for the signal-response time function of Models A (uncontrolled), B and C. These values represent the area below each curve in Supplementary Figure 2, that is, the sum of the transient times for each response. (DOC) [file pone.0031095.s005.doc]

**Supplementary tables**

**Supplementary Table 1. Overall response times for the three systems modeled (uncontrolled comparison) ^a^.**

|  | **Modulation of SK autophosphorylation (k_1_)** | | **Modulation of SKP dephosphorylation (k_2_)** | |
| --- | --- | --- | --- | --- |
|  | **OFF → ON** | **ON → OFF** | **OFF → ON** | **ON → OFF** |
| **Monofunctional** |  |  |  |  |
| Model A | 3 011.49 | 1 143.31 | 15 515.40 | 27 816.30 |
| Model B | 3 406.48 | 1 337.95 | 9 467.02 | 24 801.00 |
| Model C | 3 125.05 | 1 091.73 | 57 574.80 | 43 048.20 |
| **Bifunctional** |  | | | |
| Model A | 3 346.30 | 1 378.56 | 9 336.50 | 20 907.90 |
| Model B | 3 672.27 | 1 739.08 | 8 695.38 | 10 672.20 |
| Model C | 3 358.06 | 1 195.35 | 57 212.80 | 40 114.40 |

^a^ Results of the integral for the signal-response time function of Models A (uncontrolled), B and C. These values represent the area below each curve in Supplementary Figure 2, that is, the sum of the transient times for each response.
